# Caudal epidural anesthesia during intracavitary brachytherapy for cervical cancer

**DOI:** 10.1093/jrr/rrv011

**Published:** 2015-04-06

**Authors:** Yuko Isoyama-Shirakawa, Katsumasa Nakamura, Madoka Abe, Naonobu Kunitake, Keiji Matsumoto, Saiji Ohga, Tomonari Sasaki, Satoru Uehara, Kazuhiro Okushima, Yoshiyuki Shioyama, Hiroshi Honda

**Affiliations:** 1 Department of Radiation Oncology, National Hospital Organization Kyushu Cancer Center, 3-1-1 Notame, Minami-ku, Fukuoka 811-1395, Japan; 2 Department of Clinical Radiology, Graduate School of Medical Sciences, Kyushu University, 3-1-1 Maidashi, Higashi-ku, Fukuoka 812–8582, Japan; 3 Department of Radiation Oncology, Fukuoka Tokushukai Hospital, 4–5 Sukukita, Kasuga, Fukuoka 816–0864, Japan; 4 Ion Beam Therapy Center, SAGA-HIMAT Foundation, 415 Harakoga, Tosu, Saga 841–0071, Japan

**Keywords:** cervical cancer, intracavitary brachytherapy, caudal epidural anesthesia, pain control

## Abstract

It has been suggested that pain control during intracavitary brachytherapy for cervical cancer is insufficient in most hospitals in Japan. Our hospital began using caudal epidural anesthesia during high-dose-rate (HDR) intracavitary brachytherapy in 2011. The purpose of the present study was to retrospectively investigate the effects of caudal epidural anesthesia during HDR intracavitary brachytherapy for cervical cancer patients. Caudal epidural anesthesia for 34 cervical cancer patients was performed during HDR intracavitary brachytherapy between October 2011 and August 2013. We used the patients' self-reported Numeric Rating Scale (NRS) score at the first session of HDR intracavitary brachytherapy as a subjective evaluation of pain. We compared NRS scores of the patients with anesthesia with those of 30 patients who underwent HDR intracavitary brachytherapy without sacral epidural anesthesia at our hospital between May 2010 and August 2011. Caudal epidural anesthesia succeeded in 33 patients (97%), and the NRS score was recorded in 30 patients. The mean NRS score of the anesthesia group was 5.17 ± 2.97, significantly lower than that of the control group's 6.80 ± 2.59 (*P* = 0.035). The caudal epidural block resulted in no side-effects. Caudal epidural anesthesia is an effective and safe anesthesia option during HDR intracavitary brachytherapy for cervical cancer.

## INTRODUCTION

Intracavitary brachytherapy is an important treatment modality for gynecological cancer, but it has been pointed out that many patients suffer from severe vaginal pain and discomfort during this treatment if sufficient pain control and sedation are not achieved. It has been reported in Western countries that general, spinal [1] and epidural anesthesia, and conscious sedation with fentanyl and midazolam [2, 3] are effective for pain control in patients treated with high-dose-rate (HDR) intracavitary brachytherapy. Tsujino *et al*. [4] surveyed methods of pain control and sedation during HDR intracavitary brachytherapy for cervical cancer in Japan. Their survey revealed that general, epidural or spinal anesthesia was not performed during HDR intracavitary brachytherapy in most Japanese hospitals for varying reasons, such as a shortage of anesthetists and the risks of anesthesia. Many radiation oncologists in Japan responded that pain control during HDR intracavitary brachytherapy was insufficient for a certain percentage of patients [4].

Interstitial brachytherapy and hybrid brachytherapy consisting of intracavitary and interstitial brachytherapy are becoming more widely performed in Japan, and these procedures might need stronger pain control than intracavitary brachytherapy alone. In addition, image-guided 3D planning, which is frequently combined with brachytherapy, takes more time than 2D planning, so the control of pain during intracavitary brachytherapy is becoming more important.

Caudal epidural anesthesia is used frequently with minor complications for perianal procedures. Although caudal epidural anesthesia is the most commonly used regional anesthesia technique for children, it has also been administered to adults to control pain [5]. Caudal epidural anesthesia was reported to be one of the analgesic techniques available for managing pain in intracavitary brachytherapy of gynecological cancer [6].

We have performed caudal epidural anesthesia to reduce patients' pain during HDR intracavitary brachytherapy for gynecological cancer since 2011. The effect and safety of caudal epidural anesthesia for brachytherapy has never been reported in Japan, to our knowledge. The purpose of the present study was to retrospectively investigate the effects of caudal epidural anesthesia during HDR intracavitary brachytherapy for cervical cancer patients.

## MATERIAL AND METHODS

We performed HDR intracavitary brachytherapy for 55 patients with cervical cancer from October 2011 to August 2013 at our hospital. Of them, 34 women treated with HDR intracavitary brachytherapy using a tandem applicator were evaluated. The cases of 21 of the 55 patients were excluded from the present analysis because they did not undergo caudal epidural anesthesia: 14 patients declined the caudal epidural anesthesia; five patients did not receive the anesthesia because only ovoid applicators were used and the pain was mild, and two patients could not receive anesthesia because they had been treated with an anticoagulant drug. We did not perform caudal epidural anesthesia for patients who had a tendency to bleed, had an allergy for local analgesics or had problems at the punctured site. Full informed consent was obtained from all 34 patients.

Radiation therapy for cervical cancer was administered with a combination of external beam radiation therapy (EBRT) and HDR intracavitary brachytherapy with ^192^iridium applications. We attempted to administer caudal epidural anesthesia for 34 women with cervical cancer at every session of HDR intracavitary brachytherapy using a tandem applicator. Radiation oncologists who were trained by anesthetists administered the caudal epidural anesthesia.

**Fig. 1.**
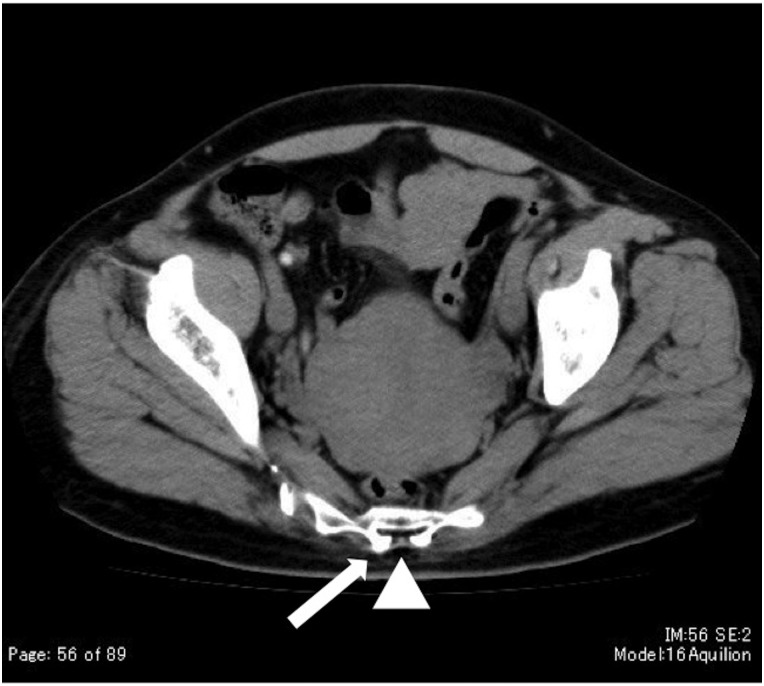
CT image of a 51-year-old woman. Arrowhead: sacral hiatus. Arrow: sacral cornua.

**Fig. 2.**
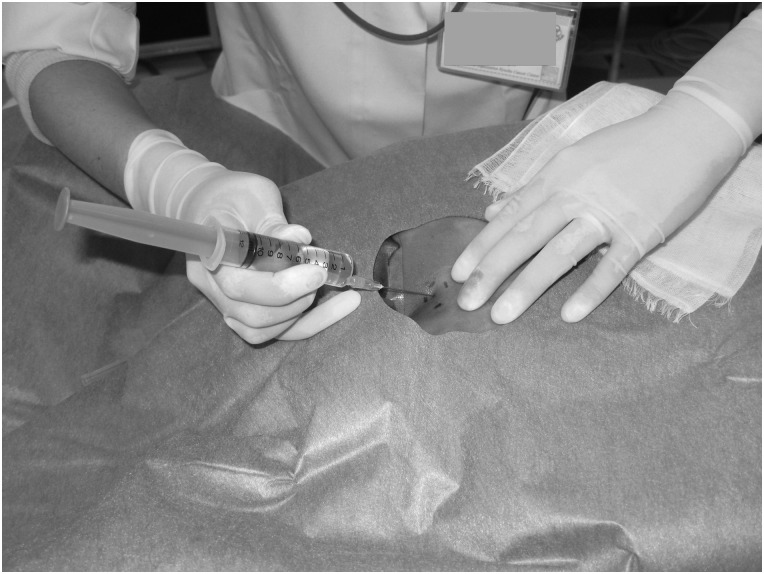
Caudal epidural anesthesia procedure. The sacral hiatus was punctured with a needle through the sacrococcygeal membrane.

We checked the form of the sacral hiatus using diagnostic computed tomography (CT) imaging to plan the external beams, before the anesthesia was initiated. The sacral hiatus of one patient on a CT image is shown in Fig. 1. As shown in Fig. 2, the patients were in the prone position and the sacral hiatus was typically identified between the sacral cornua. The sacral hiatus was punctured with a needle through the sacrococcygeal membrane, and 10 ml of 1% mepivacaine hydrochloride (1% Carbocaine®) was injected after confirming that there was no backflow of blood or spinal fluid. The patients were put in the lithotomy position, and the procedures of brachytherapy were performed. Relaxation of the anal sphincter muscle could be established by the success of the caudal epidural anesthesia. The patient's blood pressure, pulse, and oxyhemoglobin saturation by pulse oximetry were monitored during the procedures. The duration of caudal epidural anesthesia was ∼3 h, and the patients were ordered to rest in bed until 3 h after injection of mepivacaine hydrochloride.

Some drugs that had been used before starting the caudal epidural anesthesia were used concomitantly as an analgesic or sedative. Diclofenac sodium (Voltaren®) was used as a suppository in some patients, and pentazocine hydrochloride (Pentazine®) or morphine hydrochloride hydrate was administered intravenously as an analgesic. Hydroxyzine hydrochloride (Atarax®) or haloperidol was administered as a sedative in some patients before the caudal epidural anesthesia.

An 11-point Numeric Rating Scale (NRS) score [patients rate their pain from 0 (no pain) to 10 (severe pain)] at the first session of HDR intracavitary brachytherapy was used as a subjective evaluation of maximal pain during the procedures. We compared the self-reported NRS scores of the patients with caudal epidural anesthesia with that of 30 patients who underwent HDR intracavitary brachytherapy using a tandem applicator without sacral epidural anesthesia between May 2010 and August 2011 at our hospital. The patients without sacral epidural anesthesia were given only an analgesic and/or a sedative mentioned above.

The total dose of EBRT in both groups ranged from 45 to 50.4 Gy (median 50.4 Gy). The median EBRT dose to the whole pelvis was 30.6 Gy, and the median remaining dose of EBRT with a midline block was 19.8 Gy. The median total dose of HDR intracavitary brachytherapy to point A was 24 Gy in the control group and 30 Gy in the anesthesia group. The dose per fraction to point A was 6 Gy.

The χ^2^/Fisher's test was used to compare drugs used concomitantly as analgesics and sedatives between the control group and the anesthesia group. The NRS score was compared between the two groups using the Mann–Whitney U-test, and *P*-values < 0.05 were accepted as significant.

## RESULTS

In all 34 patients, the sacral hiatus was identified by the CT for planning EBRT. The NRS score was not recorded in the medical records in three of the patients. For one patient, the drug could not be injected into the extradural space because the sacral cornua was not palpated and the sacral hiatus was not identified from the body surface. The success rate of caudal epidural anesthesia was, thus, 97% (33/34).

**Fig. 3.**
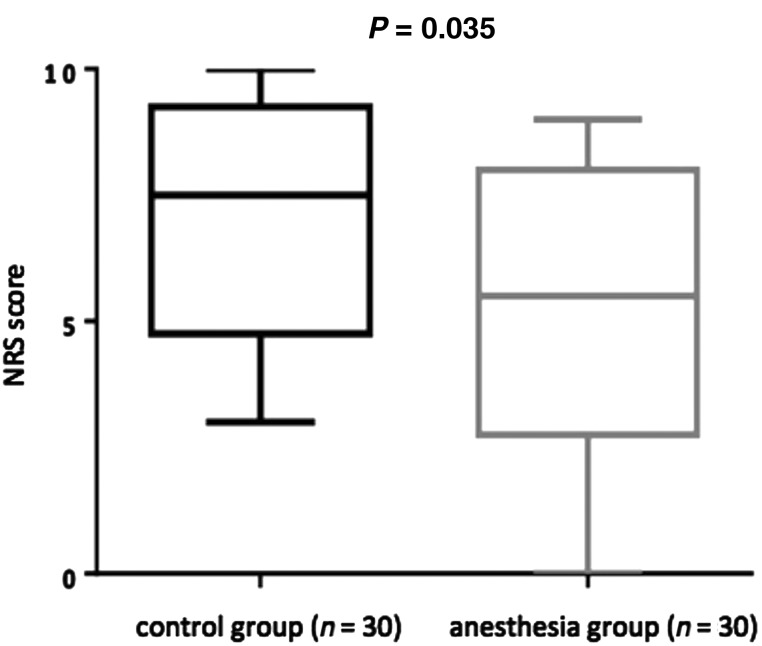
The box plots of the NRS score during HDR-ICRT. Left: the control group without caudal epidural anesthesia (*n* = 30). Right: the group with caudal epidural anesthesia (*n* = 30). The top and bottom of the box represent the third and first quartile, respectively. The middle line indicates the median.

The patient characteristics and the method of radiation therapy are shown in Table 1. There were no significant differences in the patients' ages or the brachytherapy device used between the control group and the anesthesia group. The drugs used as analgesics and sedatives (other than the sacral epidural anesthesia) are listed in Table 2. Pentazocine hydrochloride and hydroxyzine hydrochloride were used significantly more often in the control group than in the anesthesia group (*P* = 0.038 and *P* = 0.037, respectively), and opioid was used significantly more often in the anesthesia group (*P* = 0.033). The mean NRS score of the anesthesia group was 5.17 ± 2.97, significantly lower than that of the control group's mean score of 6.80 ± 2.59 (*P* = 0.035) (Fig. 3). There was no complication caused by the caudal epidural anesthesia, such as local anesthetic-induced toxicity, dural puncture, hematoma, or infection at the site of puncture. Respiratory depression and decline of blood pressure were not seen.

**Table 1.** Characteristics of cervical cancer patients and the methods of radiation therapy

		Control group	Anesthesia group
Age (years)		33–85 (median 56.5)	31–74 (median 54)
FIGO	1b	13	10
	2a	1	0
	2b	8	7
	3a	3	0
	3b	5	11
	4a	0	2
External beam radiation therapy	Dose to whole pelvis:	0–50.4 Gy (median 30.6 Gy)	0–50.4 Gy (median 30.6 Gy)
	Dose with a midline block:	0–50.4 Gy (median 19.8 Gy)	0–50.4 Gy (median 19.8 Gy)
Brachytherapy		18–30 Gy (median 24 Gy)	18–30 Gy (median 30 Gy)
Type of device	tandem + ovoid	27	28
	tandem + cylinder	3	2

FIGO = International Federation of Gynecology and Obstetrics.

**Table 2.** Analgesic and sedatives used concomitantly with the caudal epidural anesthesia

		Control group	Anesthesia group	*P*-value
Analgesic	Pentazocine hydrochloride	19	10	0.038
	Opioid	7	16	0.033
	Diclofenac sodium	6	8	n.s.
Sedatives	Hydroxyzine hydrochloride	18	9	0.037
	Haloperidol	15	18	n.s.

n.s. = not significant.

## DISCUSSION

Although intracavitary brachytherapy is an important treatment modality for cervical cancer, many patients suffer from severe vaginal pain and discomfort during treatment without anesthesia. It is reported that general, spinal [1] and epidural anesthesia, and conscious sedation with fentanyl and midazolam [2, 3] are sufficient for pain relief during HDR intracavitary brachytherapy.

According to a survey of the Gynecologic Cancer Intergroup in 2009 [7], 97% of responders' patients received pain relief for applicator insertion during brachytherapy, consisting of general (46%), spinal (27%), intravenous conscious sedation (28%), and/or oral pain medication (14%). Tsujino *et al*. [4] surveyed 144 hospitals in Japan in 2011 regarding how they control pain during HDR intracavitary brachytherapy. The survey responses indicated that general anesthesia (1%), epidural/spinal anesthesia (2%), intravenous conscious sedation and/or analgesia (36%), intramuscular and/or subcutaneous injection (22%), and oral medication and/or suppository (31%) were performed for intracavitary brachytherapy. Most of the radiation oncologists (73%) surveyed responded that pain control during brachytherapy was insufficient for patients. In particular, radiation oncologists who perform intracavitary brachytherapy without anesthesia or strong sedative analgesics such as midazolam tended to feel inadequate pain control.

The findings reported by Tsujino *et al*. [4] also revealed that most radiation oncologists avoided general/spinal/epidural anesthesia and strong sedative analgesics because the hospital's manpower was insufficient and/or they were concerned about the risks that accompany the administration of anesthesia and strong sedative analgesics.

Caudal epidural anesthesia has been used widely in obstetrics, having gained popularity in the 1940 s as a means of ensuring a painless childbirth [8], but misapplications of the technique led to failures, complications and a subsequent decline in its use. The reasons underlying the decline in the use of caudal epidural anesthesia are mainly related to variations in the anatomy of the sacral hiatus. This variation of anatomy is more common in adults than in children. Caudal epidural anesthesia is thus the most commonly used anesthesia technique for children. However, the ability to locate the hiatus and define anatomical variations is the main determinant of the success of this anesthesia technique in adults, and it can be performed with great success when there is sufficient practical experience and a knowledge of the pertinent anatomy through imaging techniques, including ultrasound [5, 9] and CT. This method is now safe and useful, even in adults, particularly for operations performed in the anal and sacral regions [10].

Caudal epidural anesthesia has some advantages over lumbar epidural anesthesia and spinal anesthesia [11]. Caudal epidural anesthesia provides more reliable perineal anesthesia. Some studies showed that as high as 21% and as few as 6.7% of lumbar epidurals fail to S1 [12]. In addition, the incidence of dural puncture is lower using a caudal rather than a spinal or lumbar epidural, because the dural sac ends at S2. The duration of anesthesia from a single-dose caudal injection is longer than that from a single-dose spinal injection.

Complications associated with caudal epidural anesthesia include local anesthetic-induced toxicity, dural puncture, hematoma, and infection at the site of puncture [10], which are the same complications as those seen with most other regional anesthesia procedures. The administration of an excessive dose of a local anesthetic and unintentional administration of a drug into an epidural vein should be avoided to prevent local anesthetic–induced toxicity [10]. We do not administer caudal epidural anesthesia for patients who are taking anticoagulant drugs to prevent hematoma. Dawkins [12] mentioned one permanent lesion in nearly 23 000 cases, but the details were not specified in that report.

The presence of ligamentous and osseous abnormalities precludes the attainment of satisfactory anesthesia in 5–10% of individuals [7]. Sekiguchi [13] reported an anatomical study of the sacral hiatus in Japanese subjects; 4% of 92 cases showed an absent sacral hiatus. At our hospital, the anatomy of the sacral hiatus is checked in CT images before the caudal epidural anesthesia is initiated, and in the present patient series the success rate of caudal epidural anesthesia was 97%. We did not observe an absent sacral hiatus in any of the 34 patients. In one patient, we could not inject the drug into the extradural space because the sacral cornua could not be palpated. Najman [5] reported that ultrasound can be a useful tool for positioning the needle in the caudal space, especially in difficult cases.

There are few reports about local anesthesia during brachytherapy for cervical cancer. Paracervical block is a commonly used local anesthetic for minor gynecological procedures. The typical placement of 10% lidocaine 4 ml on the cervix and vagina in HDR intracavitary brachytherapy has been evaluated, and a reduction in visual analogue scale (VAS) scores from 60 ± 24.8 mm to 49.9 ± 24.1 mm has been reported [14]. Chen *et al*. [15] reported the analgesic effect of local vaginal anesthesia during HDR intracavitary brachytherapy for cervical cancer. The mean VAS scores recorded during the treatment sessions and control sessions were 49.9 ± 24.1 and 60.1 ± 24.8, respectively, which is significantly lower in the treatment sessions.

Conscious sedation techniques are also useful for controlling pain during intracavitary brachytherapy. Bhanabhai *et al*. [3] reported that the maximal pain score in 57 procedures of 20 patients who underwent HDR brachytherapy for cervical cancer under conscious sedation with midazolam and an opioid ranged from 0 to 10 (median 4.7). Kwekkeboom *et al*. [16] reported that the mean NRS score of the worst procedural pain at the first session of 17 patients who underwent HDR brachytherapy under conscious sedation with midazolam and fentanyl was 3.14 ± 2.11.

In the present study, we found that the NRS score was significantly lower in the group with caudal anesthesia than in the control group (5.17 ± 2.97 vs 6.80 ± 2.59). However, the mean NRS score of the caudal epidural in this study was not as low as that in studies that used stronger conscious sedation [3, 16]. One reason is that caudal epidural anesthesia is expected to be effective for regions around the cervix and vagina, but not sufficient for a patient to tolerate the presence of applicator rods in the body of the uterus [6]. Caudal epidural anesthesia has an effect on the sacral and lower lumbar nerve roots. Distention of the cervix and upper vagina stimulates parasympathetic autonomic afferents from the pelvic splanchnic nerves of S2–S4, and vaginal packing stimulates somatic afferents via the pudendal nerves of S2–S4 [6, 17]. However, the presence of applicator rods in the body of the uterus stimulates sympathetic autonomic afferents, which enter the spinal cord at the T10–L1 level [6, 17]. Another reason for the present study groups' difference in NRS scores is that the anesthesia group's score included pain due to the insertion of a needle.

Our study has some limitations. There was a bias in that the patients who did not feel strong pain during the dilatation of the cervix by their gynecologist did not undergo caudal epidural anesthesia. There were also differences between the two groups regarding the use of drugs used concomitantly as an analgesic or sedative.

An advantage of regional anesthesia is that the method has less influence on respiration and circulation compared with strong conscious sedation. The regional anesthesia complication rate is reported to be reasonably low, and no complications occurred among the 34 patients in the present study.

## CONCLUSION

Caudal epidural anesthesia is one of the options for effective and safe regional anesthesia during HDR intracavitary brachytherapy for cervical cancer. The combination of regional anesthesia and conscious sedation was observed to reduce patient pain and discomfort during this procedure, without increasing the risk of complications.

## FUNDING

Funding to pay the Open Access publication charges for this article was provided by National Hospital Organization Kyushu Cancer Center.
